# Metabolomics for origin traceability of lamb: An ensemble learning approach based on random forest recursive feature elimination

**DOI:** 10.1016/j.fochx.2025.102856

**Published:** 2025-08-01

**Authors:** Chongxin Liu, Simona Grasso, Nigel Patrick Brunton, Qi Yang, Shaobo Li, Li Chen, Dequan Zhang

**Affiliations:** a Institute of Food Science and Technology, Chinese Academy of Agriculture Sciences, Key Laboratory of Agro-Products Quality and Safety Control in Storage and Transport Process, Ministry of Agriculture and Rural Affairs, Beijing 100193, China; b School of Agriculture and Food Science, University College Dublin, Belfield, Dublin 4, Ireland

**Keywords:** Lamb, Machine learning, Metabolomics, Origin traceability, Random forests, Recursive feature elimination

## Abstract

The origin traceability of lamb is a longstanding concern for consumers. Despite the widespread application of untargeted metabolomics in meat origin traceability, challenges remain in achieving rapid and accurate identification of biomarkers. This study utilized untargeted metabolomics to analyse five breeds of geographical indication lamb, obtaining profile data comprising a total of 4139 metabolites. Using random forest recursive feature elimination, 29 potential biomarkers were initially identified, which showed significant breed-specific and production environment-related variations. Upon further assessment, a refined panel of 14 metabolic biomarkers demonstrated optimal accuracy and robustness in tracing lamb origin. When combined with the Naive Bayes algorithm, these biomarkers yielded the highest classification accuracy among all evaluated machine learning methods. The random forest recursive feature elimination presents a practical approach for handling high-dimensional metabolomics datasets compared to previous analytical methods. These findings also provide valuable insights into the development of machine learning-based biomarker panels, greatly enhancing the breed-specific traceability of lamb.

## Highlights

• First use of metabolomics with machine learning for lamb origin authentication.
• Total of 4139 metabolites were identified in lamb samples from five sheep breeds.
• Biomarkers screened successfully using random forest recursive feature elimination.
• Data fusion from multiple ion modes improved model robustness and interpretability.
• Naive Bayes showed strong potential for food origin tracing with small samples.

## Graphical abstract

**Figure:**
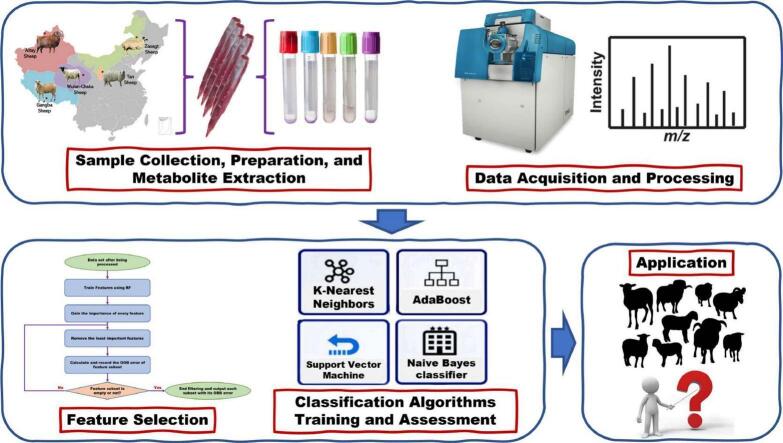

## 1 Introduction

The advancement of logistics and preservation techniques has prompted consumers to pursue a variety of global foods, especially those produced outside their local area (Kantono et al., 2021). These foods are generally considered as premium because of the special qualities associated with their geographical origin or cultural heritage and thus carry a higher price. (Camin et al., 2018; Henchion et al., 2014). Taking the example of lamb production and consumption in China, non-local lamb commands a premium of 20 % - 25 % over the local type (Li et al., 2022). This is due to higher production and transport costs, coupled with strong consumer demand. Some local suppliers may find it easier to engage in fraudulent practices within the food production and supply chain. For example, they might mislabel the origin of lamb, disguising locally bred lamb as a breed from another region in order to reduce procurement and logistics costs and increase their profits. While such practice may not pose a direct health risk, it seriously undermines consumer confidence and raises concerns about food traceability. Therefore, the development of scientific detection methods to accurately identify the origin of food is crucial for mitigating such fraudulent activities.

Metabolites, influenced by both intrinsic factors and external environmental conditions, are key indicators for discriminating food sources. The research indicates that the metabolite fingerprints of plant-based food are mainly influenced by environmental factors, including varieties, moisture, temperature, and soil minerals (McClements & Grossmann, 2021). Additionally, animal-derived food is affected by factors such as biological inheritance, age, feed composition, and slaughter season (D'Alessandro et al., 2019; Juárez et al., 2008), which contribute to the complexity of authentication assessment. Metabolomics has emerged as a powerful method for verifying multiple food sources (Wang et al., 2022; Smaoui et al., 2023; Du et al., 2023). Over the past decade, improvements in high-throughput mass spectrometry have significantly increased the number of identifiable metabolites in animal foods. For example, Zhang et al. (2022a) only identified 1705 metabolites from New Zealand lamb rib meat using rapid evaporative ionization mass spectrometry (REIMS), while Qie et al. (2022) identified 5503 metabolites (3170 in positive ion mode and 2333 in negative ion mode) from the *longissimus dorsi* muscles of four sheep breeds using high-resolution mass spectrometry. As the accuracy of mass spectrometry and the abundance of metabolites in public databases increase, the number of identified metabolites rises. This renders traditional chemometric approaches unsuitable for the analysis of vast omics datasets, requiring a greater investment of time (Cambiaghi et al., 2016). Consequently, developing data analysis methods for authenticating food origins has become a pressing research challenge (Reel et al., 2021).

In recent years, machine learning technology has garnered widespread attention from researchers across various fields due to its excellent classification and regression capabilities (Lin et al., 2022). Traditional chemometric techniques such as partial least squares (PLS) have been widely used for traceability and adulteration detection of various food products, including tea (Xia et al., 2024), wine (Rapa et al., 2023), red pepper (Zhang, Lin, et al., 2024) and salmon (Hong et al., 2023). However, these linear methods often face challenges when dealing with high-dimensional datasets. Some of the ensemble learning methods have the critical advantage of rapidly filtering irrelevant information from large datasets to extract key feature variables (Chen et al., 2020). Particularly noteworthy is that recursive feature elimination (RFE) methods which integrate support vector machines (SVM) and random forest (RF) algorithms have proven to be effective in the analysis of spectral data. For example, Sun et al. (2017) used random forest recursive feature elimination (RF-RFE) analysis of hyperspectral data to identify key wavelengths for detecting dimethoate in mixed pesticides on lettuce leaves. Their model achieved an R^2^ of 0.8712, indicating strong predictive performance. In another study, Wang et al. (2021) applied spectral data fusion to predict the storage time of infant formula. A regression model based on wavelengths selected by support vector machine recursive feature elimination (SVM-RFE) outperformed the PLS model, achieving the highest accuracy across all storage temperatures. These findings clearly highlight the methodological advantages of RF-RFE over traditional chemometric approaches, data mining accuracy, feature selection efficiency, and the ability to handle complex datasets (Rocha et al., 2020). Despite these advances, the application of RFE to metabolomics data for screening food biomarkers remains relatively underexplored (Erban et al., 2019).

The primary aim of this study is to assess the feasibility of using an ensemble learning approach based on RF-RFE to process metabolomic data for lamb origin traceability. Non-targeted metabolomic analysis was conducted on lamb samples from main sheep breeds from five different locations, employing RF-RFE to identify the most effective biomarkers under different conditions. Preliminary results indicate that RF-RFE provides a stable feature selection mechanism, as the integrated Naive Bayes (NB) models consistently achieve accuracies above 0.9 across all biomarker panels. Compared to other machine learning algorithms, NB has been shown to be well-suited for metabolomics analyses involving limited sample sizes. The study also contributes reliable foundational technical information into the development of machine learning-based biomarker panels and enhances the breed-specific traceability of lamb.

## 2 Materials and methods

### 2.1 Reagents and chemicals

Methanol and acetonitrile were purchased from Merck Chemicals GmbH (Darmstadt, Germany). Acetic acid was purchased from Yien Chemical Technology Co., Ltd. (Shanghai, China). Ammonium formate, ammonia, and formic acid were purchased from Aladdin Biochemical Technology Co., Ltd. (Shanghai, China). All reagents used were HPLC grade. Mixed standards (**Table S1**) for metabolomic analysis were purchased from Zzbio Co., Ltd. (Shanghai, China), Toronto Research Chemicals (Ontario, Canada), Tokyo Chemical Industry Co., Ltd. (Tokyo, Japan), and Sigma-Aldrich Inc. (St. Louis, USA). Additionally, the ultrapure water used in the solution preparation process was obtained using an Aquapro 3 water purification system (Aquapro International Company LLC, Delaware, USA).

### 2.2 Sample collection

In this study, all animal procedures were performed in accordance with animal welfare and ethics guidelines (ISO/TS 34700: 2016), which were approved by the Animal Care and Use Committee of the Institute of Food Science and Technology, Chinese Academy of Agricultural Sciences (IFST-2023-302). One of the most representative sheep breeds in each of the five provinces or autonomous regions in China with the highest lamb production was chosen as the subject of the study. A total of 35 sheep (7 for each breed) were selected from local breeding farms. Comprehensive information regarding the sheep breeds and their respective sources can be found in **Table S2**. It is worth noting that the sheep belonging to each breed exhibited remarkable similarity in terms of genetic backgrounds, carcass weights, and dietary patterns.

Professional workers at local commercial abattoirs slaughtered the sheep using halal methods. Then, they removed the head, feet, skin and offal sequentially in accordance with industry standards. Samples of the *longissimus dorsi* were taken from the sheep carcasses that had been stored for 24 h in a constant-temperature room at 4 °C. The fascia and fat were removed from the muscle surface using a scalpel, and muscle samples weighing approximately 1 g were placed in 2 mL cryopreservation tubes and immersed in liquid nitrogen for 60 min. The samples were transported to the laboratory on dry ice and stored at −80 °C for further experiments.

### 2.3 Metabolite extraction

The metabolite extraction method reported by Wang et al. (2023) was modified and metabolites were extracted with one technical replicate for each biological sample (Guo et al., 2024). Samples were retrieved from a − 80 °C freezer and thawed on ice. They were then homogenized in liquid nitrogen until uniformity was achieved, which took approximately 20 min. A 50 mg (±1 mg) portion of each sample was weighed into a numbered 0.5 mL centrifuge tubes (3810×, Eppendorf, Wesseling-Berzdorf, Germany). Then, 400 μL of a methanol-water solution (methanol:water = 7:3, *V*/V) containing an internal standard mix was added. The mixture was vortexed using a Vortex mixer (VORTEX-5, Kylin-Bell, Haimen, China) at 1500 rpm for 5 min, cooled in an ice bath for 15 min and finally centrifuged at 12000*g* and 4 °C for 10 min using a centrifuge (5424R, Eppendorf, Hamburg, Germany). Following the transfer of 300 μL of the supernatant to a new centrifuge tube, the tube was left to stand for 30 min at −20 °C. Thereafter, the tube was subjected to centrifugation for 3 min at 12,000*g* and 4 °C. Subsequently, 200 μL of the supernatant was transferred to a vial lined with an insert for subsequent liquid chromatograph mass spectrometry (LC-MS) analysis.

### 2.4 Metabolomics data acquisition

After metabolite extraction, the samples were divided equally into two portions. Each portion was analysed using ultra-high performance liquid chromatography coupled with triple time-of-flight mass spectrometry/mass spectrometry (UHPLC-TripleTOF-MS/MS) in either positive or negative ion mode. The UHPLC Nexera LC-30 A system (Shimadzu, Japan) was used with an ACQUITY Premier HSS T3 column (1.8 μm, 2.1 mm × 100 mm, Waters, Milford, MA, USA). The elution gradient was modified according to the procedure reported by Zhang, Sun, et al. (2024), with a mobile phase consisting of a 0.1 % formic acid aqueous solution (mobile phase A) and a 0.1 % formic acid-acetonitrile solution (mobile phase B). The gradient elution and analysis conditions were identical for both modes, with the following specific parameters: 0–2 min with 5 % - 20 % mobile phase B, 2–5 min with a linear increase of mobile phase B from 20 % to 60 %, 5–6 min with an increase of mobile phase B from 60 % to 99 %, holding at this state for 1.5 min, followed by a transition to 5 % mobile phase B within 0.1 min and held for 2.4 min. The sample injection volume was 4 μL, with a flow rate of 0.4 mL/min, and the column temperature was maintained at 40 °C.

Metabolites were analysed using TripleTOF-MS/MS (6600+, SCIEX Foster City, CA, USA). The ion source parameters were set as follows: ion source gas 1 (GAS1) at 50 psi, ion source gas 2 (GAS2) at 50 psi, curtain gas (CUR) at 25 psi, temperature (TEM) at 550 °C, declustering potential (DP) set at 60 V or − 60 V in positive or negative mode respectively, and ion spray voltage floating (ISVF) set at 5000 V or − 4000 V in positive or negative mode respectively. The TOF-MS scan parameters were set as follows: mass range was set to 50–1000 Da, accumulation time was set to 200 ms, and dynamic background subtraction was turned on. The product ion scan parameters were set as follows: mass range was set between 25 and 1000 Da, accumulation time was 40 ms, collision energy was set at 30 V or − 30 V in positive or negative mode respectively, collision energy spread was 15, resolution was set to unit, charge state was 1:1, intensity was 100 cps, isotopes within 4 Da were excluded, mass tolerance was 20 mDa, and a maximum of 18 candidate ions were monitored per cycle. The data acquisition was carried out using the Analyst TF 1.7.1 software (Sciex, Concord, ON, Canada) in information-dependent acquisition (IDA) mode. Quality control (QC) samples prepared by equal volumes of all samples were performed to remove unacceptable characteristic peaks, resulting in a higher quality metabolomics dataset (Zhao et al.,2020).

### 2.5 Data processing

According to the method reported by Zhao et al. (2020), the initial data files obtained by LC-MS were converted into mzXML format using ProteoWizard software (Version 3.0.24081.29e5468, http://proteowizard.sourceforge.net). Peak extraction, alignment, and retention time correction were then performed using the XCMS program. To ensure the accuracy of the experimental data and the robustness of the experimental design, the integrity of the data was evaluated by discarding peaks with a detection rate under 50 % directly and filtering the missing values of the other peaks using the k-nearest neighbour (KNN) method in each group of samples. The support vector regression (SVR) method was used to correct the peak area. Identification of metabolite information was performed by retrieving the integrated public database, the AI database, metDNA, and the self-built database from the laboratory. Public database used include Metlin (http://metlin.scripps.edu/index.php), HMDB (https://hmdb.ca/), KEGG (https://www.kegg.jp/), Mona (https://mona.fiehnlab.ucdavis.edu/) and MassBank (http://www.massbank.jp/). Metabolites were considered acceptable when the relative standard deviation (RSD) of the peak area was below 30 %, the Q1 mass error remained within 25 ppm, and the deviation in retention time did not exceed 0.1 min.

### 2.6 Data analysis

#### 2.6.1 Descriptive statistics

The data processed by the XCMS program were first assessed using principal component analysis (PCA) and then used for further analysis. PCA was performed using MetaboAnalyst 6.0 (https://www.metaboanalyst.ca/) and the results are presented in the supplementary data (**Fig. S1**). Hierarchical cluster analysis (HCA) was generated using Metware Cloud platform (https://cloud.metware.cn/) to characterise metabolite differences among different lamb breeds. Bar plots were performed to compare the number of metabolites and metabolic types identified under both positive and negative ion modes.

#### 2.6.2 Selection of biomarker features

The sheep breeds, Altay sheep (AS), Gangba sheep (GS), Tan sheep (TS), Wulan-Chaka sheep (WS), and Zasagt sheep (ZS), were treated as different categories. The peak areas of all identified metabolites from both positive and negative ion modes were used as the predictive features. A RFE approach based on the RF algorithm was applied to identify the key biomarker features among all identified metabolites from the metabolomic dataset. The R packages used for RF-RFE analysis were varSelRF and caret (Acharjee et al., 2016). The varSelRF package was used to identify the most important variables and calculate the out-of-bag (OOB) error for the metabolomics dataset. The caret package was used to reduce the number of features in the metabolomics dataset.

Before analysing, the maximum feature proportion of metabolites randomly selected as candidates at each split was identified by grid searching with values ranging from 0.1 to 0.9. The 10-fold cross-validation result showed that the maximum feature proportion value of 0.4 had the optimal RF classification accuracy (**Table S2**). The other hyperparameters of RF-RFE were optimised by grid search (tidymodels package) prior to analysis, with the following settings:

• The dataset was randomly divided into two subsets, designated as the training set and the test set, with a ratio of 7:3.
• A 10-fold cross-validation procedure was used to evaluate the predictive performance of the models.
• The Gini index was employed as the criterion for evaluating node splitting.
• The number of decision trees (ntree) was 500.
• The minimum number of samples required for the internal node splitting process was 2.
• The minimum number of samples for leaf nodes was 1.
• The minimum weight of samples in leaf nodes was 0.
• The maximum depth of the tree was 10.
• The maximum number of leaf nodes was 50.
• The threshold value for the degree of impurity in the node division was 0.

**Fig. 1:**
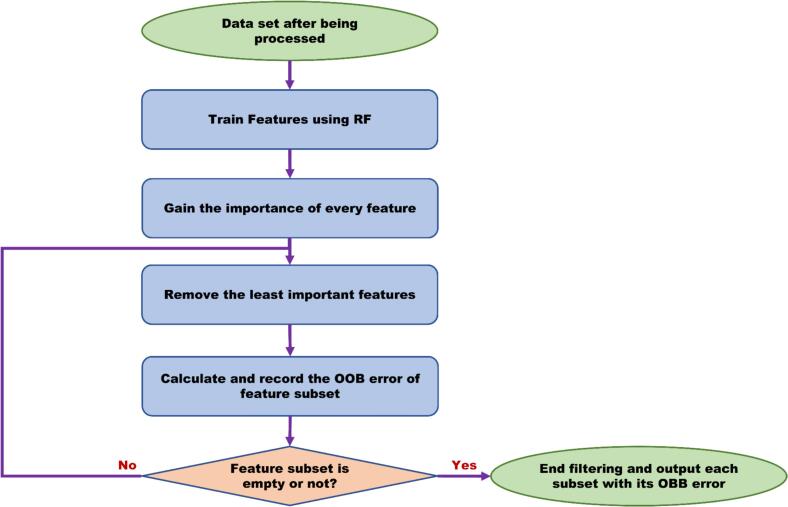
The flowchart of the random forest recursive feature elimination analysis.

As shown in Fig. 1, the RF analyses were conducted on each dataset to rank the importance of metabolite profiles based on the majority vote of each feature. Each group of features performed 30 parallel tests during RF analysis. The OOB errors for multiple tests are reported as mean ± standard deviation (SD), which is used as the final indicator of biomarker selection. The analyses were repeated after removing the bottom 20 % of metabolite profiles until it was no longer possible to repeat the process (Jeon & Oh, 2020).

#### 2.6.3 Receiver operating characteristic curve analysis

The receiver operating characteristic (ROC) curves were plotted to analyse the sensitivity and specificity of each marker screened in the different modalities. The independent predictive and diagnostic efficacy of each marker in predicting the origin of lamb in the different modalities was further assessed by comparing the area under curves (AUC). AUC values ranged from 0.5 to 1.0. The closer AUC value was to 1 the higher prediction accuracy. Generally, the predictive accuracy was low when the AUC value was between 0.5 and 0.7, somewhat accurate when the AUC value was between 0.7 and 0.9, and highly accurate when the AUC value was above 0.9. When the AUC value was equal to 0.5, the biomarker was deemed to have no effect on predicting the occurrence of the event and was therefore of no predictive value (Wang et al., 2024).

#### 2.6.4 Classification algorithms training and assessment

Four kinds of machine learning methods, KNN, Adaptive Boosting (AdaBoost), SVM, and NB, were used as classification methods to predict the origin of lamb samples based on selected metabolic biomarkers. The analysis processes for all methods were performed using the R programming language, and the hyperparameters of each method were also optimised using grid search with the following specific settings:

• KNN: The number of nearest neighbours (k) is 5, and the leaf size is 30. The search algorithm is a brute force approach, the nearest neighbours sample weight function is uniform, and the vector distance algorithm is Euclidean.
• AdaBoost: The number of base classifiers is 100, and the learning rate of the classifier is 1.
• SVM: The penalty factor is set to 10,000. The kernel function is radial basis function (RBF), the kernel function factor is scale, the kernel function constant is 0, the kernel function maximum number of terms is 3, the error convergence condition is 0.001, and the maximum number of iterations is 1000. The multi-classification fusion strategy is OVR.
• NB: The prior distribution is of a polynomial nature. The alpha is set to a default value of 1. The threshold for sample feature binarization is 0.

All the classification models of the four methods were cross-validated on samples using 10-fold cross-validation. The accuracy and robustness of the machine learning methods were evaluated by the results from both cross-validation and the test set. All algorithms utilized cross-validation procedures to assess the robustness of the results and the generalization ability of the models.

The differences in classification performance were assessed comprehensively in four aspects: Accuracy, Recall, Precision, and F1 Score. The specific calculated equations are as follows:

(1) $$\mathrm{Accuracy}=\frac{\mathrm{TP}+\mathrm{TN}}{\mathrm{TP}+\mathrm{FP}+\mathrm{TN}+\mathrm{FN}}$$

(2) $$\mathrm{Recall}=\frac{\mathrm{TP}}{\mathrm{TP}+\mathrm{FN}}$$

(3) $$\mathrm{Precision}=\frac{\mathrm{TP}}{\mathrm{TP}+\mathrm{FP}}$$

(4) $$F1\;\mathrm{Score}=\frac{2*\mathrm{Precision}*\mathrm{Recall}}{\mathrm{Precision}+\mathrm{Recall}}$$

where *TP* (True Positive) indicates the correctly predicted positive example; *TN* (True Negative) indicates the correctly predicted negative example; *FP* (False Positive) indicates the incorrectly predicted positive example; *FN* (False Negative) indicates the incorrectly predicted negative example.

The assessment indicators are interpreted based on the criteria according to Liu et al. (2024). The results under different modes were also used to compare these four classification methods. Confusion matrices for all test sets were also presented to compare the performance differences between different biomarkers and machine learning methods used for classification.

## 3 Results and discussion

### 3.1 Descriptive statistics of metabolite profiles

**Fig. 2:**
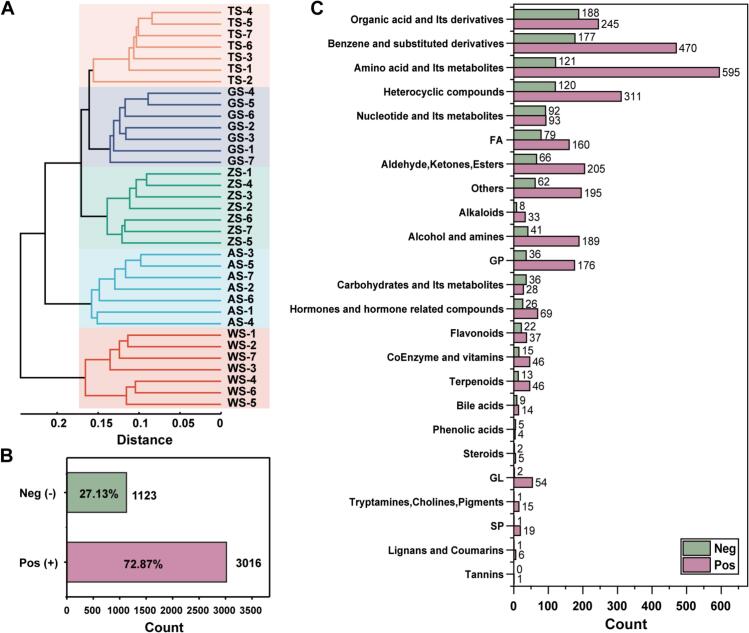
Descriptive statistical analyses of metabolomics data for five lamb breeds, including (A) hierarchical clustering analysis of lamb samples, (B) the number and percentage of metabolites in negative and positive ion modes, and (C) the number of metabolites in different categories. (Note: FA, Fatty Acids; GP, Glycerophospholipids; GL, Glycerolipids; SP, Sphingolipids.)

As shown in Fig. 2, the metabolite profiles of five lamb samples from the *longissimus dorsi* were assessed by UHPLC-TripleTOF-MS/MS. The results of the HCA indicate that the complete metabolite profiles were successful in distinguishing the origin of the samples (Fig. 2A), and some of the metabolite abundances differed obviously between the breeds (**Fig. S2**). However, due to the extensive number of metabolites, it is challenging to directly identify the metabolites that could be effectively used for discrimination from the heatmap alone (**Fig. S2**). Further screening was required to eliminate most of the low-importance features.

A total of 4139 metabolites were identified after qualitative analysis and filtering based on MS2 and metabolomics databases, of which 3016 metabolites were identified in positive ion mode and 1123 metabolites were identified in negative ion mode (Fig. 2B). All of these identified metabolic compounds were categorized into 24 classes, with the 10 classes having the highest number of metabolites being amino acids and their metabolites (716), benzene and substituted derivatives (647), organic acids and their derivatives (433), heterocyclic compounds (431), aldehydes, ketones, esters (271), fatty acids (239), alcohols and amines (230), glycerophospholipids (212), nucleotides and their metabolites (185), and other unclassified substances (Fig. 2C). Notably, this finding aligns closely with the results reported by Hu et al. (2024), which demonstrated a general enrichment of amino acid metabolites in animal muscle tissue. This phenomenon may be attributed to the central structural and functional role of proteins in myocytes. As essential components for cell growth and repair, proteins form the structural foundation of myocytes. In addition, their degradation products play important roles in various physiological and biochemical processes, such as cell signalling, energy metabolism, and the stress response.

It is important to note that other classes of metabolites may also be important and should not be disregarded. For example, a study that employed direct analysis in real time high-resolution mass spectrometry to compare the metabolite profiles of four distinct lamb breeds revealed that carbohydrates, including *N*-acetyl-d-glucosamine and D(−)-fructose, as well as hormone compounds, including thromboxane B2, can be utilized as effective biomarkers for differentiating between lamb sources (Qie et al., 2022). In addition, lipids, as one of the major nutrients in animals, are involved in the physiological activities and metabolic processes of the body. Liu et al. (2024) found lipids showed significant differences between Tan sheep and Bahan crossbreed sheep through quantitative lipidomics, in which glycerophospholipids and glycerolipids were proven to be the main lipid biomarker classes used to identify the meat from Tan sheep. Therefore, further screening is required to identify key metabolic markers for source determination of lamb.

### 3.2 Potential metabolic biomarkers screening

To further assess the differences between metabolites from different lamb samples, differential metabolite screening was performed using various metabolic profiles as datasets to identify potential metabolite markers for origin differentiation of lamb based on the RF-RFE method. The metabolomic dataset was randomly split, with 70 % of the data belonging to the training set and 30 % to the test set. Specifically, during the construction of classification trees using the RF algorithm, the training dataset is created using bootstrap sampling. This means that 70 % of the samples from the original dataset are randomly sampled with replacement to construct multiple subsets of the training data.

However, to evaluate model performance, a portion of independent data is needed for testing to verify the model's performance on unseen data. Therefore, the OOB error estimation is used for performance evaluation on the screened set of marker data. Approximately 30 % of the samples remain at random and are not included in any of the bootstrap samples. These excluded samples are referred to as OOB data and can be used to test the performance of the classification tree, as they are never used to train the model. By comparing the predicted results of these OOB samples with the actual labels, the OOB error, an unbiased estimate of the classification performance of the classification tree on unseen data, can be calculated.

The procedure for biomarker screening using the RF-RFE method is detailed in Supplementary Data **Table S3**. As observed in a previous study (Phan & Tomasino, 2021), the OOB error demonstrates a fluctuating trend as the number of feature values diminishes throughout the screening process. This phenomenon can be attributed to the fact that RFE eliminates features in descending order of significance. When it begins to eliminate features of lesser importance, the impact on the model's performance is minimal, and in some cases, it may even enhance the model's performance. Consequently, the OOB error declines. However, when continuing to remove features, it is advisable to gradually remove those that are critical to the model's performance. When these critical features are removed, the predictive power of the model is reduced, resulting in an increase in the OOB error (Xia & Yang, 2022).

**Fig. 3:**
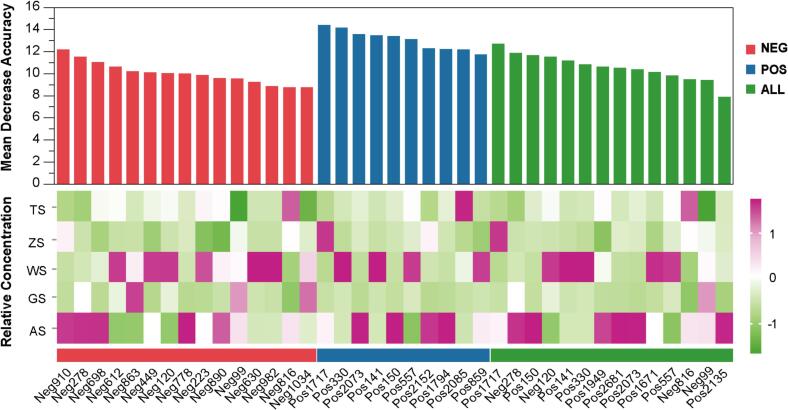
Ranking and differences in abundance of metabolic markers using the RF-RFE method in negative ion mode, positive ion mode and all metabolite data. (Note: Relative concentration data were *Z*-score normalised before heat map plotting.)

When the number of characteristic variables was reduced to fewer than 20, the most significant metabolites were identified based on the lowest OOB error rates across different models. Specifically, 15 metabolites were selected in negative ion mode, 10 in positive ion mode, and 14 from the full metabolic profiles. The major metabolites dataset selected according to the above constraints provides a good balance between low OOB % and a low number of metabolites. Accordingly, Fig. 3 shows the importance ranking and abundance differences of metabolic markers using the RF-RFE method in different modes.

### 3.3 Identification and reliability analysis of selected metabolic markers

Table 1 lists these important metabolites screened along with their chemical formulas, retention times, and calibration mass-to-charge ratio information used for metabolite identification. Similar to the results of Chen et al. (2023b), more than half of the identified metabolite classes screened for metabolites important in classifying the sources of lamb included fatty acids, amino acids and their metabolites, benzene and substituent derivatives, organic acids and their derivatives, nucleotides and their metabolites, and glycerophospholipids (GP).

**Table 1:** Potential metabolic markers to identify the lamb breeds.

Metabolite ID	Metabolite name	Class	Adduct	Formula	Retention Time (min)	Exact mass (*m*/*z*)	Mass Error (ppm)
***Neg (−)***							
Neg99	2-Methyl-6-phytylquinol	Benzene and substituted derivatives	[M-H]-	C_27_H_46_O_2_	6.3850	402.3498	9.0197
Neg120	L-Tryptophan	Amino acid and Its metabolites	[M-H]-	C_11_H_12_N_2_O_2_	2.4831	204.0899	0.1915
Neg223	Inosine	Nucleotide and Its metabolites	[2 M-H]-	C_10_H_12_N_4_O_5_	1.7796	268.0808	0.1945
Neg278	L-Cysteine	Amino acid and Its metabolites	[M + CH_3_COO]-	C_3_H_7_NO_2_S	0.8143	121.0197	1.3236
Neg449	Sedoheptulose 7-phosphate	Carbohydrates and Its metabolites	[M-H]-	C_7_H_15_O_10_P	0.8606	290.0403	1.0790
Neg612	Benzeneacetonitrile	Benzene and substituted derivatives	[M-H]-	C_8_H_7_N	2.4831	117.0578	0.3750
Neg630	L-Aspartyl-*L*-phenylalanine	Amino acid and Its metabolites	[M-H]-	C_13_H_16_N_2_O_5_	2.5870	280.1059	1.6803
Neg698	Maleic acid	Organic acid and Its derivatives	[M-H]-	C_4_H_4_O_4_	0.8808	116.0110	1.2166
Neg778	17-Octadecynoic acid	FA	[M-H]-	C_18_H_32_O_2_	6.2817	280.2402	0.7316
Neg816	PC(20:5(5Z,8Z,11Z,14Z,17Z)/22:0)	GP	[M-H_2_O-H]-	C_50_H_90_NO_8_P	8.7664	863.6404	17.8896
Neg863	Gln-Leu	Amino acid and Its metabolites	[M + CH_3_COO]-	C_11_H_21_N_3_O_4_	1.8663	259.1532	1.4106
Neg890	PtdIns-(1,2-dioctanoyl)	Organic acid and Its derivatives	[M-H]-	C_25_H_47_O_13_P	6.6957	586.2754	22.5232
Neg910	1-NBD-decanoyl-2-decanoyl-sn-Glycerol	Others	[M-H]-	C_29_H_46_N_4_O_8_	6.4883	578.3316	16.1468
Neg982	7-[(1*R*,2*R*,3*S*,5*S*)-2-[(4*S*)-4-(2,3-dihydro-1H-inden-2-yl)-4-hydroxybut-1-enyl]-3-fluoro-5-hydroxycyclopentyl]hept-5-enoic acid	Hormones and hormone related compounds	[M-H]-	C_25_H_33_FO_4_	2.1301	416.2363	21.2133
Neg1034	Avermectin B2b aglycone	Aldehyde, Ketones, Esters	[M-H + CH_3_CN]-	C_33_H_48_O_9_	6.3850	588.3298	0.2387

***Pos (+)***							
Pos141	Enoxacin Sesquihydrate	Others	[M]+	C_30_H_40_F_2_N_8_O_9_	2.8983	694.2886	9.2974
Pos150	Carnitine C14:1	FA	[M]+	C_21_H_39_NO_4_	5.7150	369.2879	2.5777
Pos330	Ser-Leu	Amino acid and Its metabolites	[M + H]+	C_9_H_18_N_2_O_4_	1.6703	218.1267	0.1500
Pos557	Dimetridazole	Heterocyclic compounds	[M + NH_4_]+	C_5_H_7_N_3_O_2_	2.3972	141.0538	8.1350
Pos859	LPC(18:1/0:0)	GP	[M + H]+	C_26_H_52_NO_7_P	7.1298	521.3560	1.1610
Pos1671	3’-O-Methyladenosine	Nucleotide and Its metabolites	[M-2H + 3Na]+	C_11_H_15_N_5_O_4_	2.0580	281.1124	10.5000
Pos1717	Carnitine ph-C1	FA	[M]+	C_14_H_19_NO_4_	2.6307	265.1314	2.2071
Pos1794	Carnitine C14:2	FA	[M]+	C_21_H_37_NO_4_	5.4527	367.2723	2.0969
Pos1949	Lys-Ile-Asn-Glu	Amino acid and Its metabolites	[M + H]+	C_21_H_38_N_6_O_8_	6.6720	502.2751	3.2462
Pos2073	Carnitine C18:1-OH	FA	[M + H-H_2_O]+	C_25_H_47_NO_5_	6.2488	441.3454	1.5933
Pos2085	Ala-Val-Lys	Amino acid and Its metabolites	[M]+	C_14_H_28_N_4_O_4_	3.4962	316.2111	1.0937
Pos2135	Dihydromethanophenazine	Alcohol and amines	[M]+	C_37_H_52_N_2_O	7.0076	540.4080	8.3347
Pos2152	Lys-His-Ala-Val-Ser	Amino acid and Its metabolites	[M]+	C_23_H_40_N_8_O_7_	6.4604	540.3020	8.0706
Pos2681	MG(0:0/20:3(11Z,14Z,17Z)/0:0)	GL	[M + NH_4_]+	C_23_H_40_O_4_	6.1547	380.2927	4.1497

**Note:** FA, Fatty Acids; GP, Glycerophospholipids; GL, Glycerolipids.

It is well established that the metabolite composition of sheep of different breeds is primarily influenced by genetic differences, dietary, and environmental factors. Regarding fatty acids, GP, and glycerolipids (GL), sheep belonging to the Tibetan lineage (GS and WS) exhibited distinct tail shapes compared to other Kazakh (AS) and Mongolian (TS and ZS) breeds. This distinction is attributed to Tibetan sheep living in cold, high-altitude environments for extended periods. These breeds have adapted by accumulating subcutaneous fat or by increasing oxidative metabolism to sustain body temperature. Consequently, their lipid metabolite levels are generally lower than those of other breeds (Xu et al., 2023). Inosine and 3’-*O*-methyladenosine confirmed the effect of genetic differences on metabolic differences in different breeds of sheep. Dimetridazole is a commonly used antiparasitic agent in livestock and poultry. In some regions, it may still be present in commercial feed or pharmaceutical formulations, leading to bioaccumulation in animal tissues (Xu et al., 2022). This anthropogenic factor has positioned dimetridazole as a “shadow marker”, which reflects geographic variation despite its non-natural origin. While its role as an independent traceability biomarker remains debated, it may still hold analytical value as part of a broader fingerprint that includes other endogenous metabolites.

Additionally, several crucial cellular metabolic pathway differential metabolites were identified, such as sedoheptulose 7-phosphate, an intermediate metabolite of the pentose phosphate pathway. This metabolite is synthesized by the enzyme transketolase and converted to downstream products by transaldolase. It is involved in nucleotide synthesis, aromatic amino acid synthesis, and energy metabolism. This pathway is significant for cellular reductive metabolism and oxidative stress (Rashida & Laxman, 2021).

Several studies have used the RF algorithm to screen for differential biomarkers but have often neglected reliability analysis of the selected biomarkers (Chu et al., 2024; Phan & Tomasino, 2021). The results of RF tests are characterized by randomness and incompleteness, which is crucial for constructing subsequent machine learning discrimination models (Iranzad & Liu, 2024). Therefore, ROC analyses were performed on all selected metabolites to assess the reliability of the biomarkers and the RF-RFE method.

**Fig. 4:**
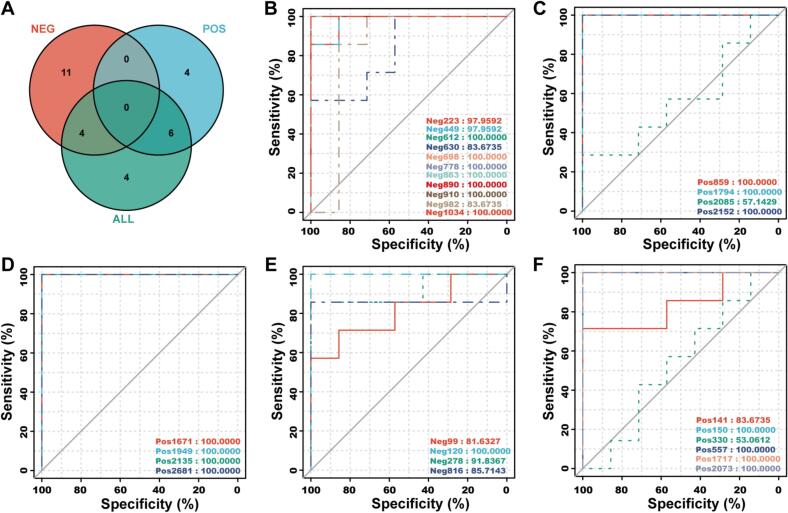
Venn diagram (A) and receiver operating characteristic curves (B—F) of metabolic markers screened by negative ion mode data, positive ion mode data and all data. (B, metabolic markers only in NEG group; C, metabolic markers only in POS group; D, metabolic markers only in ALL group; E, metabolic markers both in NEG and ALL group; F, metabolic markers both in POS and ALL group).

Venn diagram analysis classified a total of 29 metabolites screened across different modes into five distinct groups. The ROC curves are presented in Fig. 4. Among these metabolites, 19 exhibited an AUC value of 1.0, 3 had AUC values between 0.9 and 1.0, 5 AUC values between 0.7 and 0.9, and 2 AUC values between 0.5 and 0.7. An approximate AUC value of 0.5 indicates that the feature in question did not provide any independently valid information in the classification task. In other words, the ability of the dipeptide (Ser-Leu) and the tripeptide (Ala-Val-Lys) to discriminate between the five lamb sources was no better than random guessing. This suggests that these peptides, as common metabolites in sheep during physiological metabolism, lack discriminatory power for this purpose.

This is because metabolomics data usually contains a large number of invalid and noisy features. However, RF are highly resistant to interference and are usually less sensitive to missing values and noise. This indirectly leads to the selection of these noisy features out of the feature selection process. It also manifests the strong overfitting resistance of the RF algorithm (Iranzad & Liu, 2024). Furthermore, retaining some noise features can help prevent overfitting when evaluating machine learning algorithms used for classification. This approach also simplifies the complexity of subsequent analytical steps.

### 3.4 Integrated machine learning techniques for lamb origin identification

Traditional machine learning techniques are commonly used to identify fraud in high-value food products. Several studies have integrated these methods with foodomics data, demonstrating success in cases such as wine (Phan & Tomasino, 2021), cereals (Chen et al., 2023), and eggs (Chin et al., 2023). In this study, four machine learning algorithms based on different principles (KNN, AdaBoost, SVM, NB) were selected to further analyse the screened biomarkers to classify the sources of lamb. To ensure the accuracy and reliability of the evaluation results, cross-validation was employed to reduce the impact of chance. This involves training and testing different subsets of data multiple times, thereby increasing the robustness of the evaluation process. The confusion matrices and detailed classification results of the multi-class discrimination models under different conditions on the test set are shown in **Fig. S3** and Table 2, respectively. All results from the four methods showed acceptable accuracy values (greater than 0.6). However, there were noticeable differences in the classification results when using metabolites screened under different modes. The combinations of these screened markers with the SVM and NB algorithms were more accurate in identifying the source of lamb than the combinations with KNN and AdaBoost. For example, in negative mode, the prediction accuracy on the test set was, in descending order, NB (90.9 %), SVM (90.9 %), AdaBoost (81.8 %), and KNN (72.7 %), with the cross-validation set showing a similar trend. In addition, the metabolic biomarkers screened by integrating all profiles showed higher accuracy and robustness in identifying lamb sources.

**Table 2:** Comparison of prediction results by integrating KNN, AdaBoost, SVM and NB algorithms.

Method	KNN			AdaBoost			SVM			NB		
Mode	Neg (−)	Pos (+)	All (±)	Neg (−)	Pos (+)	All (±)	Neg (−)	Pos (+)	All (±)	Neg (−)	Pos (+)	All (±)
***Cross-validation***												
Accuracy	0.600	0.817	0.950	0.850	0.933	0.967	0.750	0.900	0.967	0.967	1.000	1.000
Recall	0.600	0.817	0.950	0.850	0.933	0.967	0.750	0.900	0.967	0.967	1.000	1.000
Precision	0.667	0.850	0.925	0.900	0.967	0.944	0.692	0.900	1.000	0.967	1.000	1.000
F1 Score	0.622	0.828	0.933	0.867	0.944	0.953	0.711	0.900	0.978	0.967	1.000	1.000

***Test***												
Accuracy	0.727	0.818	0.909	0.818	0.909	1.000	0.909	1.000	1.000	0.909	1.000	1.000
Recall	0.727	0.818	0.909	0.818	0.909	1.000	0.909	1.000	1.000	0.909	1.000	1.000
Precision	0.745	0.891	0.939	0.864	0.939	1.000	0.955	1.000	1.000	0.932	1.000	1.000
F1 Score	0.717	0.795	0.909	0.824	0.903	1.000	0.915	1.000	1.000	0.906	1.000	1.000

The accuracy of machine learning algorithms depends on the type of dataset being applied and the tuning of the hyperparameters, which challenges their ability to generalize in practical applications. Among these four machine learning methods, KNN and NB are straightforward and commonly used to handle multi-classification problems, but the prediction accuracies of the two methods are quite different. The discrepancy can be attributed to the fact that, despite its resilience to data noise, KNN is susceptible to underperformance when confronted with imbalanced and high-dimensional data. In contrast, NB demonstrates superior performance in the context of high-dimensional data (Wickramasinghe & Kalutarage, 2020). The markers selected by the RF-RFE approach were analysed using the original data without dimensionality reduction. Under these conditions, the KNN algorithm exhibited substantially lower predictive accuracy than the NB model.

Theoretically, Adaboost, SVM and NB all perform well on small-sized datasets, meaning that variations among their results are probably due to their different sensitivities to noisy features and the risk of overfitting the model (Rodríguez-Bermúdez et al., 2018; Sanz et al., 2018; Wickramasinghe & Kalutarage, 2020). The results of the study showed that the AdaBoost algorithm only achieves perfect accuracy results when using biomarkers screened by all metabolite data. Due to its sensitivity to noise and overfitting (Khan et al., 2024), AdaBoost is unsuitable for applications where high classification accuracy is critical. Both the SVM and NB algorithms achieve high accuracy (greater than 0.9) when classifying different pattern metabolic markers. However, the cross-validation accuracy of SVM model achieved only 0.75 on negative pattern markers, likely due to overfitting. This indicates that the classification hyperplane is overly complex, fitting noise in the training data and failing to generalize to unseen samples (Abdullah & Abdulazeez, 2021; Yang & Dong, 2019).

Data quality has a direct impact on the ability of the model to recognise complex biological patterns, which in turn affects its predictive performance on new datasets. Although there was an overall high-quality data in this study, the limited number of samples may have restricted the accuracy of the assessment and the generalisability of the model. It is important to note that although the issue of category balance was adequately addressed during the sampling phase, unbalanced distributions between categories are often observed in practical metabolomics research. In the context of high-dimensional data, failure to address category imbalance during model training may result in overfitting to majority classes, thereby neglecting critical information from minority classes. To mitigate this issue, the application of balanced sampling techniques such as Synthetic Minority Over-sampling Technique (SMOTE) or Adaptive Synthetic Sampling (ADASYN) during model development can mitigate performance degradation (Mujahid et al., 2024). The RF-RFE-based feature selection strategy effectively identified relevant features in high-dimensional metabolomic data when screening and modelling high-dimensional metabolomic data with similar structures. This strategy is not only applicable to the classification of lamb breeds but also has potential for broader application in different food categories. To further improve the stability and generalization of the proposed method, future studies should aim to increase the sample size across different food matrices, thereby improving statistical power and general applicability.

## 4 Conclusion

Variations in breed, geographical origin, and feeding conditions significantly influenced lamb quality, as evidenced by distinct alterations in metabolite profile. This study presents a practical approach for verifying food sources, which was successfully used to analyse samples from five different lamb breeds. The predictive performance of 14 metabolic biomarkers was validated using ROC curve analysis and further integrated multiple machine learning algorithms. Notably, the NB achieved the highest classification accuracy among all evaluated algorithms, underscoring its suitability for metabolomics-based analysis with limited sample sizes. The results highlight the strong potential of integrating high-throughput metabolomics with machine learning as a reliable solution for lamb traceability. The RF-RFE method also offers a powerful tool of handling complex metabolomic data which could be easily extend to other types of food. While the findings are encouraging, broader validation using diverse food types and larger-scale datasets will be essential to confirm the generalizability of the proposed approach. Future research should also focus on evaluating the stability of metabolite markers under various storage conditions to further enhance model robustness and practical applicability.

## CRediT authorship contribution statement

**Chongxin Liu:** Writing – review & editing, Writing – original draft, Visualization, Methodology, Investigation, Formal analysis, Data curation. **Simona Grasso:** Writing – review & editing, Supervision, Conceptualization. **Nigel Patrick Brunton:** Writing – review & editing, Supervision. **Qi Yang:** Investigation. **Shaobo Li:** Writing – review & editing, Software, Resources. **Li Chen:** Writing – review & editing, Supervision, Project administration, Data curation, Conceptualization. **Dequan Zhang:** Writing – review & editing, Supervision, Resources, Project administration.

## Declaration of competing interest

The authors declare that they have no known competing financial interests or personal relationships that could affect the work described in this article.

## Data Availability

Data will be made available on request.

## Appendix A Supplementary data

Supplementary material Image 1
