# Supplementary material for: Metabolomics for origin traceability of lamb: An ensemble learning approach based on random forest recursive feature elimination
Source: Food Chem X. 2025 Aug 1;29:102856. doi: 10.1016/j.fochx.2025.102856 (PMC12341576; doi:10.1016/j.fochx.2025.102856)
Supplement: Supplementary file 1 — Supplementary material [file mmc1.docx]

**Metabolomics for origin traceability of lamb: an ensemble learning approach based on random forest recursive feature elimination**

Chongxin Liu ^a,b^, Simona Grasso ^b,*^, Nigel Patrick Brunton ^b^, Qi Yang ^a^, Shaobo Li ^a^, Li Chen ^a,*^, Dequan Zhang ^a^

*^a^ Institute of Food Science and Technology, Chinese Academy of Agriculture Sciences, Key Laboratory of Agro-Products Quality and Safety Control in Storage and Transport Process, Ministry of Agriculture and Rural Affairs, Beijing 100193, China;*

*^b^ School of Agriculture and Food Science, University College Dublin, Belfield, Dublin 4, Ireland.*

*Corresponding authors*

***Simona Grasso ^*^***

*E-mail: simona.grasso@ucd.ie*

*Website: https://people.ucd.ie/simona.grasso*

***Li Chen^*^***

*E-mail:* *chenliwork@126.com*

*Website:* *https://ifst.caas.cn/en/scientists/associateprofessors/262487.htm*

**Table S1** Information on internal standard substances.

| Mode | Compounds | CAS | Q1 (Da) | RT (min) | CV | Company |
| --- | --- | --- | --- | --- | --- | --- |
| pos | L-Methionine-methyl-D3 | 13010-53-2 | 153.0764 | 0.83 | 0.06 | Zzbio Co., Ltd. |
| pos | Octanoic acid-13C | 200-813-2 | 146.1251 | 6.26 | 0.09 | Toronto Research Chemicals |
| pos | L-Tryptophan-d5 | 62595-11-3 | 210.1289 | 2.27 | 0.10 | Tokyo Chemical Industry Co., Ltd. |
| pos | L-Leucine-D7 | 92751-17-2 | 139.1455 | 1.08 | 0.10 | Sigma-Aldrich Inc. |
| neg | Octanoic acid-13C | 200-813-2 | 144.1119 | 6.26 | 0.02 | Zzbio Co., Ltd. |
| neg | benzoic acid d5 | 1079-02-3 | 126.0614 | 4.46 | 0.03 | Toronto Research Chemicals |
| neg | 2-Amino-3-(2-Chlorophenyl) Propanoic Acid | 103616-89-3 | 198.0332 | 2.61 | 0.07 | Tokyo Chemical Industry Co., Ltd. |
| neg | Citric acid-2,2,4,4-d4 | 147664-83-3 | 195.0451 | 0.80 | 0.08 | Sigma-Aldrich Inc. |

**Table S2** Information on breeds, locations, and feed conditions of lamb samples.

| Breed | Sample Size | Location | Latitude and Longitude | Altitude | Age | Sex | Raising Methods |
| --- | --- | --- | --- | --- | --- | --- | --- |
| Altay Sheep | N=7 | Fuhai County, Altay Region, Xinjiang Uygur Autonomous Region | N：47°07′5.42″  E：87°30′25.90″ | 948 m | 6 Month | Male | Fresh forage, forage silage, maize silage |
|  | | | | | | | |
| Wulan-Chaka Sheep | N=7 | Wulan County, Haixi Mongol and Tibetan Autonomous Prefecture, Qinghai Province | N：36°50′54.75″  E：98°53′54.18″ | 2986 m | 24 Month | Male | Fresh forage |
|  | | | | | | | |
| Gangba Sheep | N=7 | Sakya County, Shigatse City, Xizang Autonomous Region | N：29°15′42.85″  E：88°52′59.03″ | 4316 m | 24 Month | Male | Fresh forage |
|  | | | | | | | |
| Tan Sheep | N=7 | Yanchi County, Wuzhong City, Ningxia Hui Autonomous Region | N：37°48′10.26″  E：107°26′44.52″ | 1341 m | 6 Month | Male | Forage silage, maize silage, concentrate feed |
|  | | | | | | | |
| Zasagt Sheep | N=7 | Ulanhot, Xing'an League, Inner Mongolia Autonomous Region | N：46°17′3.76″  E：120°48′16.49″ | 269 m | 6 Month | Male | Fresh forage, forage silage |

**Table S2** Selection of maximum feature proportions (hyperparameters) for sample splitting in random forest analysis.

| Maximum Feature Proportion at Split | Neg (-) | | Pos (+) | | All (±) | |
| --- | --- | --- | --- | --- | --- | --- |
|  | Mean (%) | SD (%) | Mean (%) | SD (%) | Mean (%) | SD (%) |
| 0.1 | 92.22 | 4.04 | 90.83 | 4.56 | 90.56 | 5.84 |
| 0.2 | 93.89 | 6.47 | 92.78 | 7.20 | 94.72 | 5.42 |
| 0.3 | 92.22 | 3.60 | 90.83 | 7.36 | 92.78 | 6.89 |
| **0.4** | **94.17** | **3.46** | **93.06** | **4.27** | **95.28** | **3.40** |
| 0.5 | 93.33 | 5.87 | 91.11 | 6.47 | 94.44 | 6.55 |
| 0.6 | 93.06 | 4.00 | 91.67 | 5.68 | 90.28 | 6.87 |
| 0.7 | 90.28 | 7.33 | 90.56 | 4.30 | 94.72 | 4.00 |
| 0.8 | 92.50 | 4.80 | 92.50 | 4.31 | 90.28 | 7.41 |
| 0.9 | 92.78 | 6.55 | 92.78 | 5.84 | 94.44 | 5.44 |

**Note:** The group in bold was selected as the hyperparameter.

**Table S3** The process of RF-RFE analysis for metabolic biomarkers in breeds of lamb with different modes of data collection.

| Number of Variables | Neg (-) | |  | Number of Variables | Pos (+) | |  | Number of Variables | All (±) | |
| --- | --- | --- | --- | --- | --- | --- | --- | --- | --- | --- |
|  | Mean (%) | SD (%) |  |  | Mean (%) | SD (%) |  |  | Mean (%) | SD (%) |
| 1123 | 5.28 | 4.42 |  | 3016 | 5.70 | 4.62 |  | 4139 | 4.31 | 4.23 |
| 842 | 4.45 | 3.55 |  | 2262 | 4.72 | 4.39 |  | 3104 | 4.03 | 3.47 |
| 632 | 2.08 | 3.52 |  | 1697 | 3.75 | 4.84 |  | 2328 | 2.64 | 3.13 |
| 474 | 2.37 | 2.78 |  | 1273 | 2.92 | 3.07 |  | 1746 | 2.92 | 3.75 |
| 356 | 1.12 | 2.14 |  | 955 | 1.81 | 2.98 |  | 1310 | 2.91 | 3.74 |
| 267 | 0.98 | 1.78 |  | 716 | 1.67 | 2.55 |  | 983 | 2.23 | 2.99 |
| 200 | 0.98 | 2.07 |  | 537 | 0.84 | 1.99 |  | 737 | 1.53 | 2.95 |
| 150 | 0.56 | 1.43 |  | 403 | 0.98 | 2.07 |  | 553 | 0.42 | 1.65 |
| 113 | 0.84 | 1.99 |  | 302 | 0.84 | 1.99 |  | 415 | 0.84 | 1.99 |
| 85 | 0.42 | 1.65 |  | 227 | 0.14 | 0.75 |  | 311 | 0.56 | 1.78 |
| 64 | 0.28 | 1.05 |  | 170 | 1.39 | 2.49 |  | 233 | 0.56 | 1.43 |
| 48 | 0.28 | 1.05 |  | 128 | 1.26 | 2.20 |  | 175 | 0.28 | 1.05 |
| 36 | 0.42 | 1.26 |  | 96 | 0.98 | 1.78 |  | 131 | 0.70 | 1.57 |
| 27 | 0.42 | 1.26 |  | 72 | 1.12 | 2.14 |  | 98 | 1.96 | 2.35 |
| 20 | 0.98 | 1.78 |  | 54 | 0.70 | 1.57 |  | 74 | 1.26 | 1.92 |
| **15** | **1.40** | **1.98** |  | 41 | 0.42 | 1.26 |  | 56 | 0.56 | 1.78 |
| 11 | 2.51 | 2.55 |  | 31 | 2.38 | 2.34 |  | 42 | 0.84 | 1.68 |
| 8 | 5.01 | 3.78 |  | 23 | 2.24 | 2.10 |  | 32 | 1.68 | 2.32 |
| 6 | 4.18 | 4.82 |  | 17 | 2.10 | 2.10 |  | 24 | 2.37 | 2.57 |
| 5 | 8.61 | 5.26 |  | 13 | 2.52 | 2.06 |  | 18 | 1.95 | 2.58 |
| 4 | 6.39 | 3.34 |  | **10** | **1.96** | **2.35** |  | **14** | **1.25** | **2.44** |
| 3 | 10.13 | 4.90 |  | 8 | 2.80 | 2.72 |  | 11 | 2.09 | 2.58 |
| 2 | 10.41 | 5.13 |  | 6 | 3.34 | 2.92 |  | 8 | 1.26 | 2.68 |
|  |  |  |  | 5 | 2.79 | 2.49 |  | 6 | 3.07 | 2.83 |
|  |  |  |  | 4 | 3.48 | 2.86 |  | 5 | 1.95 | 2.79 |
|  |  |  |  | 3 | 8.06 | 4.91 |  | 4 | 3.34 | 3.11 |
|  |  |  |  | 2 | 12.36 | 5.10 |  | 3 | 5.98 | 4.88 |
|  |  |  |  |  |  |  |  | 2 | 12.92 | 5.31 |

**Note:** The groups in bold were selected for further analysis and model discrimination.


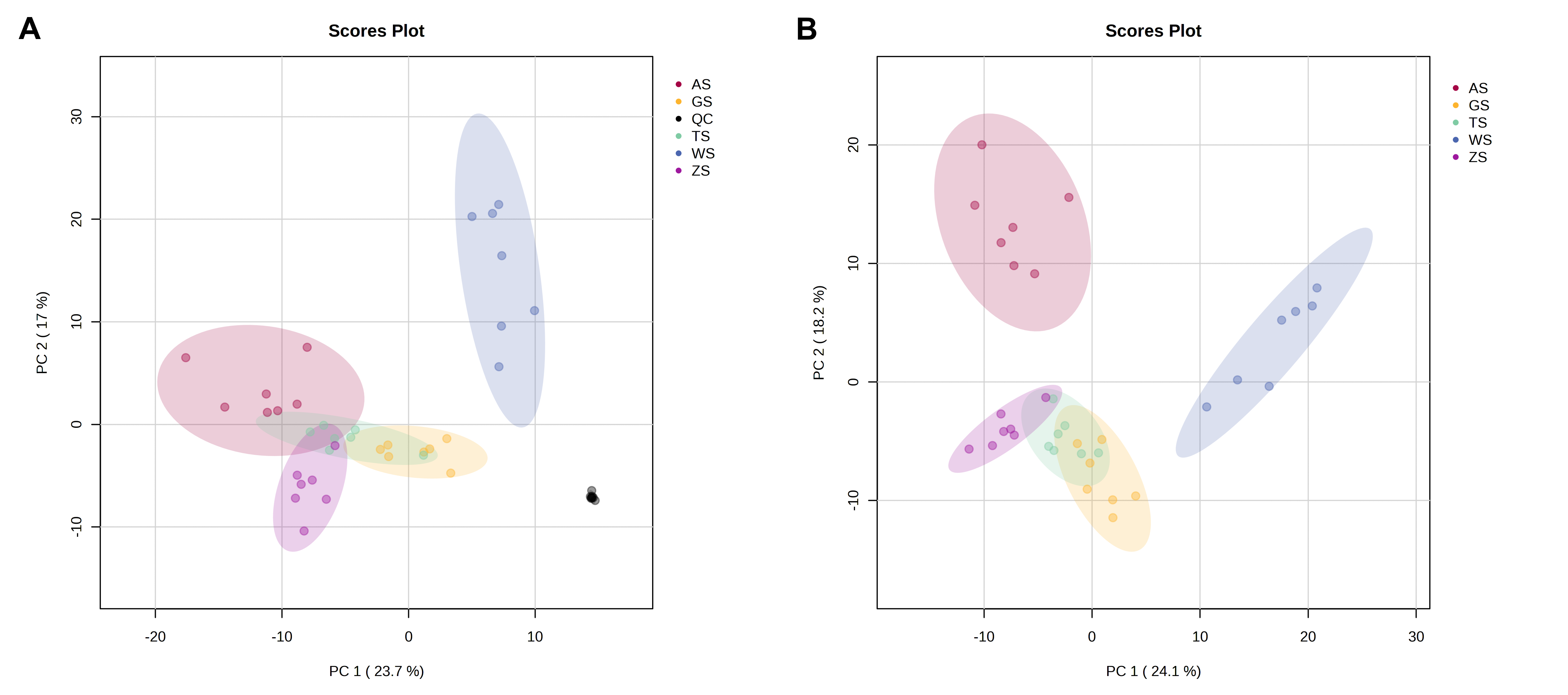
**Fig. S1.** Principal component analysis results for sample quality assessment.


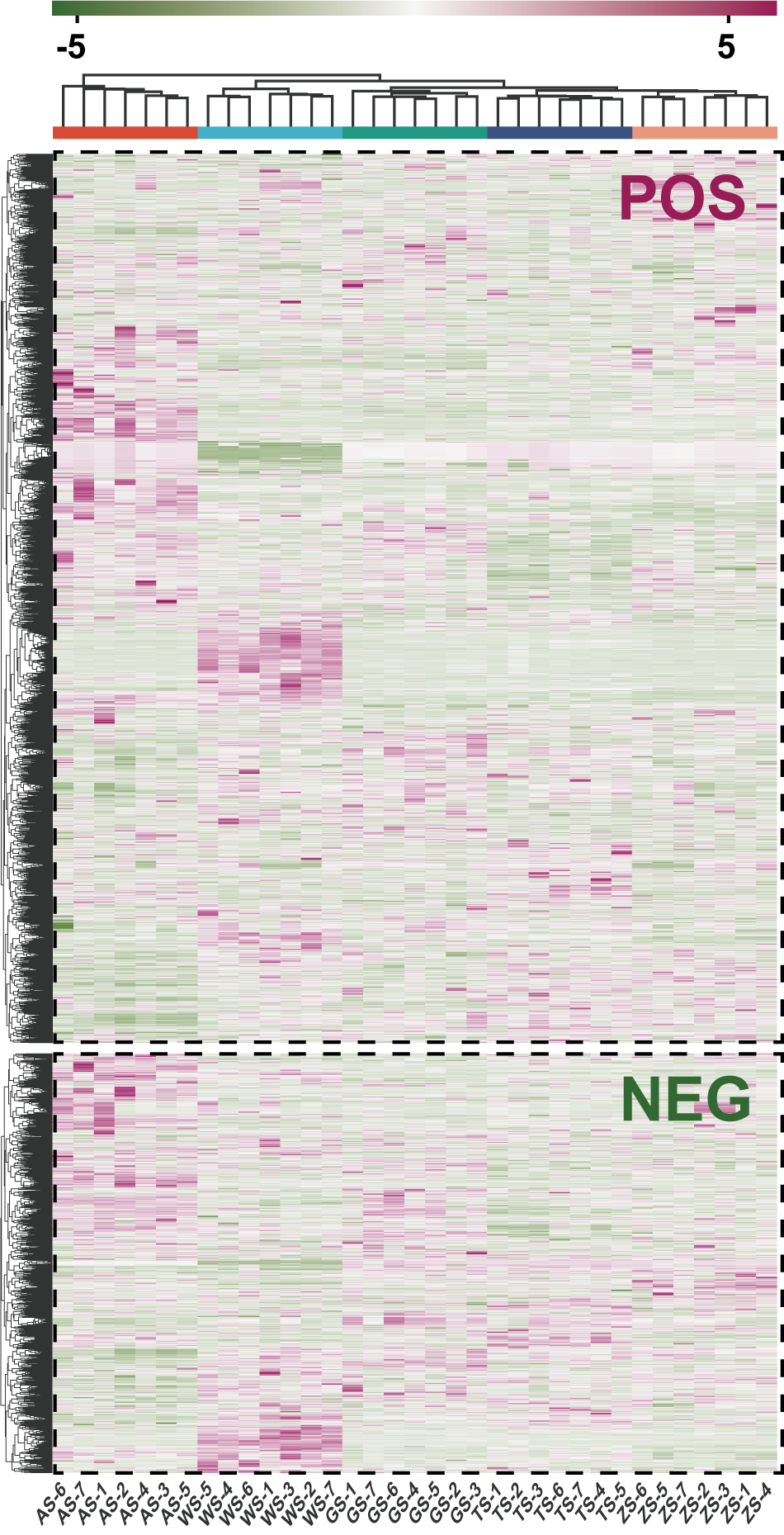
**Fig. S2.** Heatmap of metabolite profiles from samples of five lamb breeds.


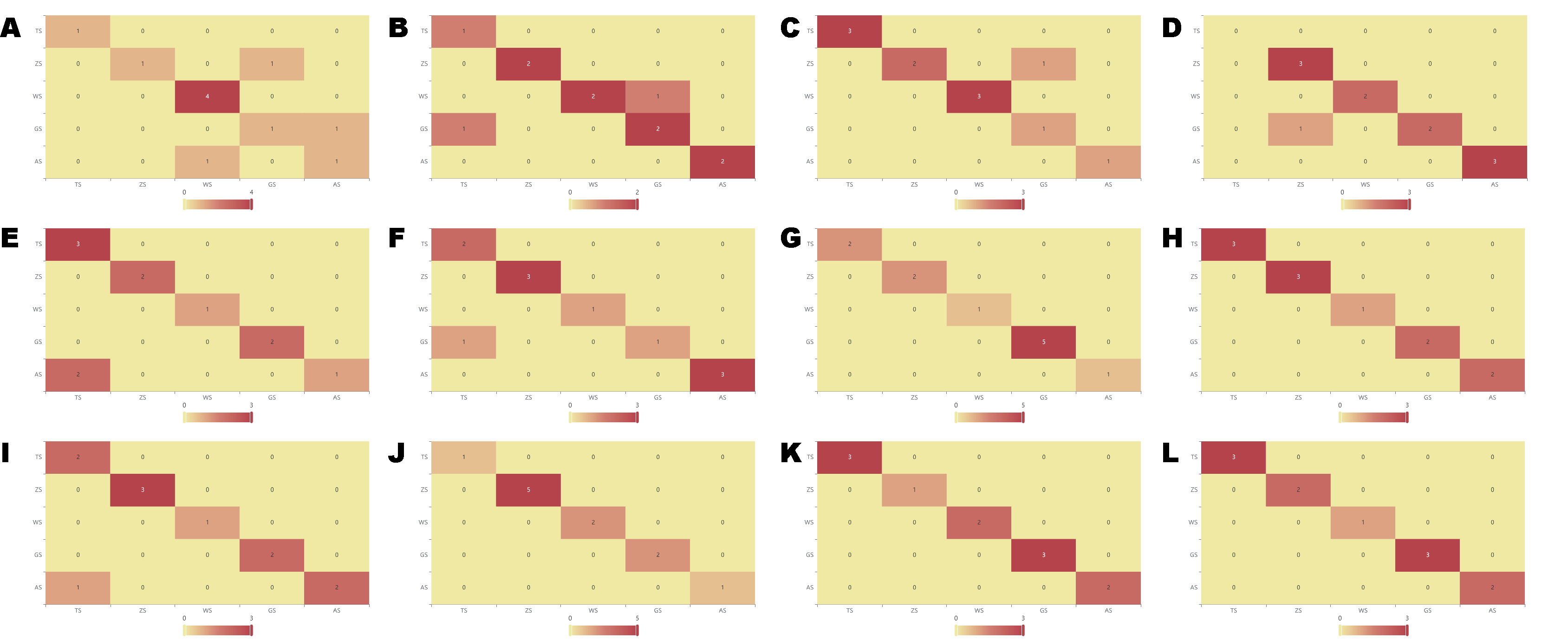
**Fig. S3.** The confusion matrices of the multi-class discrimination models under different modes (Neg: A-D; Pos: E-H; All: I-L) and machine learning algorithms (KNN: A, E, I; AdaBoost: B, F, J; SVM: C, J, K; NB: D, H, L) on the test set.
